# A Retrospective Study on Predicting Ki-67 Expression in Esophageal Cancer Patients Based on Delta Radiomics

**DOI:** 10.3390/jimaging12080346

**Published:** 2026-07-31

**Authors:** Taiwei Sun, Lei Xue, Tingting Li, Shisuo Du, Anning Cao, Yang Shen, Bei Lv, Weixing Ji, Ze Wang

**Affiliations:** 1 Department of Radiation Oncology, Zhongshan Hospital, Fudan University, 180 Fenglin Road, Shanghai 200032, China; sun.taiwei@zs-hospital.sh.cn (T.S.);; 2 Department of Thoracic Surgery, The Second Affiliated Hospital of Naval Medical University (Changzheng Hospital), 415 Fengyang Road, Shanghai 200003, China

**Keywords:** esophageal cancer, Ki-67, delta radiomics, Random Forest

## Abstract

Background: Ki-67 is a pivotal biomarker of tumor proliferative activity in esophageal cancer, yet its clinical application is hindered by reliance on invasive biopsy. Radiomics offers a non-invasive alternative, but conventional methods may be confounded by inter-individual baseline variations. This exploratory study aims to develop a radiomics-based biomarker for predicting Ki-67 expression. Methods: This single-center retrospective study included 59 patients with esophageal cancer. Delta-radiomics features were derived from preoperative CT images by calculating the difference between radiomic features from the tumor and paired normal esophageal tissue. Feature selection (mRMR, k = 3) was nested within leave-one-out cross-validation (LOOCV) to prevent data leakage. A Random Forest model was compared with Logistic Regression and Support Vector Machine across three feature types, five Ki-67 thresholds, and clinical variables. SHAP analysis was used for interpretability. Results: The Random Forest model achieved an AUC of 0.643 (95% CI: 0.483–0.792). Delta radiomics outperformed esotarget (AUC = 0.546) and eso (AUC = 0.514) models. The combined model (AUC = 0.619) did not outperform delta radiomics alone. SHAP analysis identified GrayLevelVariance and SmallAreaEmphasis as the most influential features. Conclusions: This exploratory study demonstrates that delta radiomics provides moderate discriminatory performance for predicting Ki-67 expression. External validation in independent multi-center cohorts is required before clinical application.

## 1. Introduction

Esophageal cancer is a malignant digestive tract tumor with high incidence and mortality rates worldwide, and radiotherapy plays a crucial role in its comprehensive management [1]. Currently, treatment response is primarily assessed through follow-up imaging such as computed tomography (CT) or magnetic resonance imaging (MRI), which rely on measurements of morphological changes, most commonly tumor size [2]. This approach, however, has significant limitations: imaging changes often lag behind actual biological alterations, and it remains difficult to reliably distinguish viable tumor tissue from post-radiotherapy fibrosis or necrosis [3,4,5]. Therefore, the identification of biomarkers that can provide an early, objective, and quantitative reflection of treatment efficacy is essential for advancing precision radiotherapy in esophageal cancer.

The proliferation marker Ki-67 (an antigen identified by monoclonal antibody Ki-67) is a nuclear protein expressed throughout the active cell cycle (G1, S, G2, M) but absent in quiescent cells (G0 phase); it is widely regarded as a crucial biomarker for assessing tumor proliferative activity [6,7]. Strong evidence links the Ki-67 index to tumor proliferation, invasion, metastasis, and poor prognosis in various malignancies [8]. More importantly, Ki-67 expression is sensitive to radiotherapy [9,10,11], indicating its potential as a dynamic monitoring indicator that may reflect treatment response earlier and more directly than conventional imaging. However, determining Ki-67 expression typically relies on invasive biopsy and subsequent immunohistochemical analysis, which poses a major obstacle to its routine and repeated clinical application. The key challenge lies in developing a non-invasive method to repeatedly evaluate Ki-67 expression for treatment monitoring.

Radiomics, which involves high-throughput extraction and analysis of quantitative features from medical images, can deeply mine information on intratumoral heterogeneity and has shown considerable promise in tumor diagnosis, efficacy evaluation, and prognosis prediction [12]. Studies have confirmed that radiomics can serve as a powerful tool for predicting Ki-67 expression in tumors such as breast and lung cancer [13,14,15]. Notably, recent evidence indicates that quantitative imaging parameters derived from dual-energy computed tomography (DECT) show potential for the non-invasive assessment of Ki-67 status in esophageal cancer [16], and elevated Ki-67 expression has been consistently associated with poorer survival outcomes, underscoring its prognostic relevance [17].

However, conventional radiomics analyses typically focus solely on the tumor region of interest, neglecting comparison with surrounding normal tissue, which may be influenced by inter-individual baseline variations [18,19]. To address this limitation, we propose a delta-radiomics approach, which involves calculating the differences in features between tumor and paired normal tissue. This method is designed to correct for baseline variation and more accurately reflect tumor-specific biological behaviors. To date, the application of a delta-radiomics approach based on preoperative CT to predict Ki-67 expression in esophageal cancer has not been previously reported, underscoring the novelty of our study.

Therefore, this study innovatively leverages paired radiomics features derived from tumor and normal esophageal tissue on preoperative CT images to construct a delta-radiomics signature. By establishing a link between these imaging features and Ki-67 expression status, this exploratory study aims to develop a non-invasive, radiomics-based biomarker for predicting Ki-67 expression. Future potential applications may include non-invasive, imaging-driven monitoring of treatment efficacy in esophageal cancer, pending validation in prospective multi-center studies.

## 2. Materials and Methods

### 2.1. Patient Population

This single-center retrospective study was approved by the ethics committee (see Declarations). Through collaboration with the Department of Thoracic Surgery, preoperative non-contrast chest CT images, Ki-67 immunohistochemistry results, and gastroscopy reports were collected for eligible patients. Patients with pathologically confirmed esophageal cancer who underwent a preoperative chest CT between March 2020 and December 2022 were initially screened. After rigorous clinical and imaging data review, 59 patients were included.

**Figure 1:**
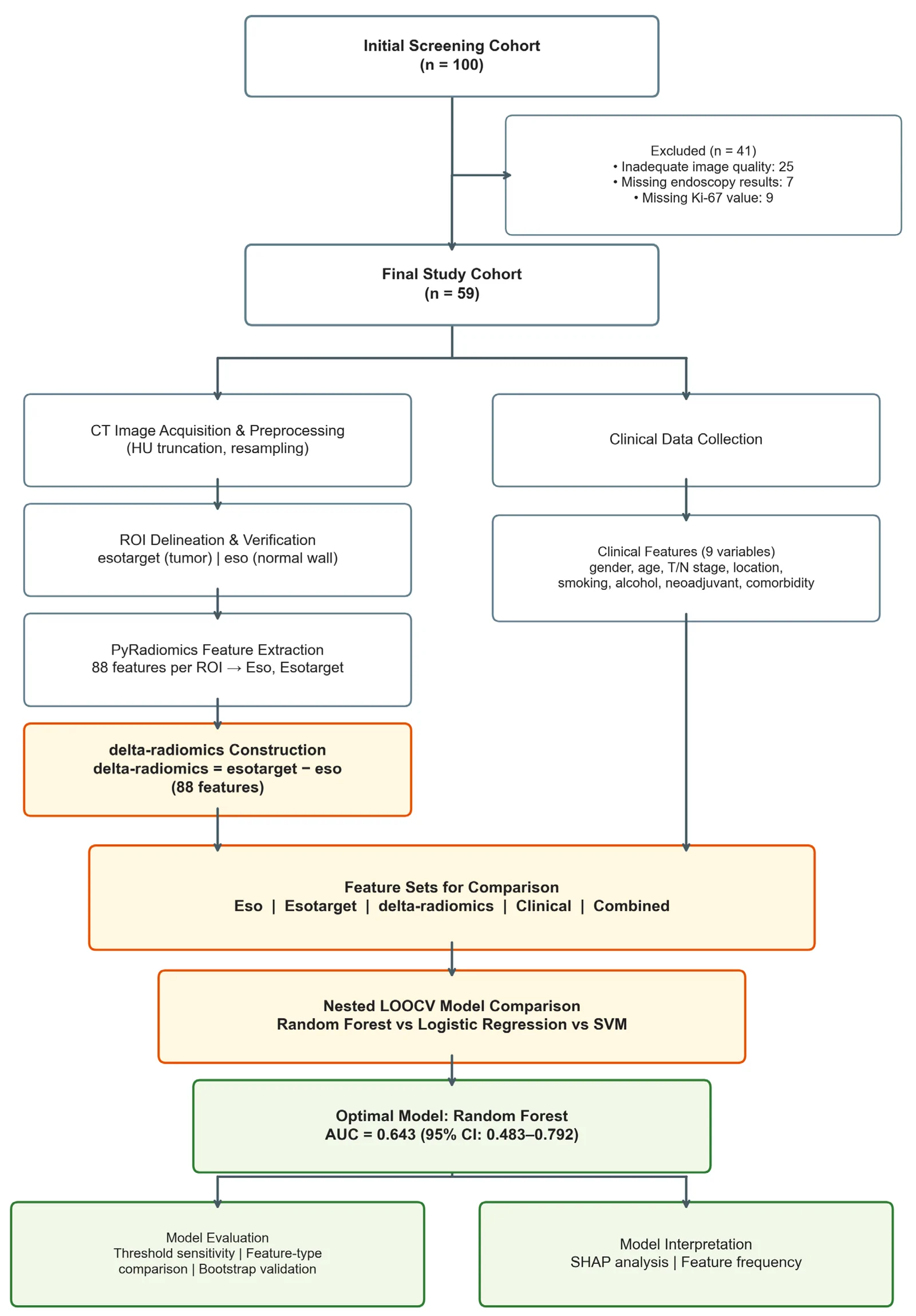
Comprehensive study flowchart integrating data collection process.

Inclusion criteria were: (1) pathological confirmation of esophageal cancer; (2) availability of preoperative chest CT images in DICOM format; (3) complete Ki-67 immunohistochemistry results; and (4) gastroscopy reports specifying tumor location. Exclusion criteria included: (1) poor image quality or severe artifacts affecting region of interest (ROI) delineation, and (2) history of prior esophageal surgery or radiotherapy. The overall workflow is illustrated in Figure 1.

**Figure 2:**
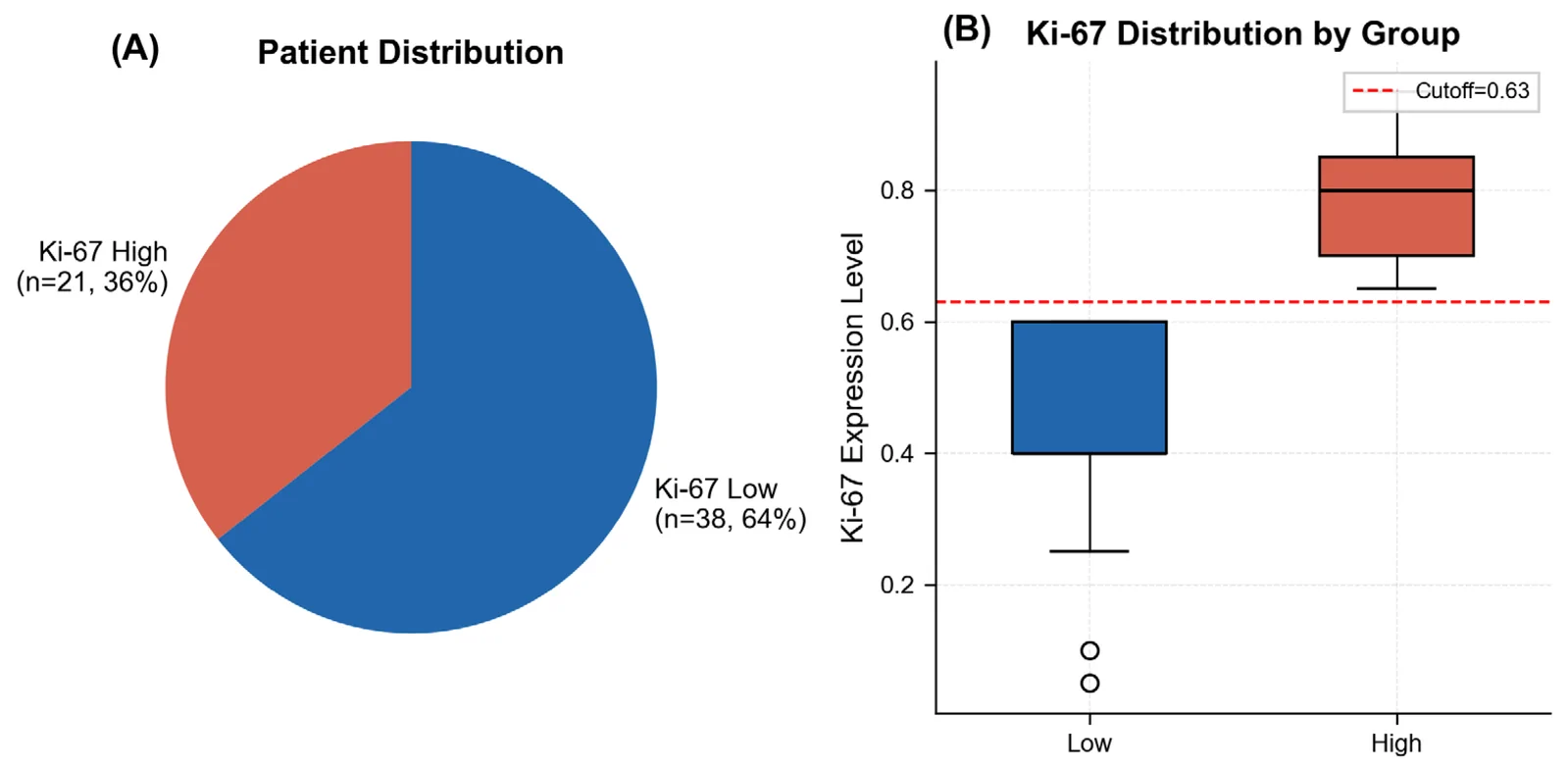
Patient cohort stratification based on Ki-67 expression. (**A**) Pie chart illustrating the proportion of patients classified into the Ki-67-high (*n* = 21, 36%) and Ki-67-low (*n* = 38, 64%) expression groups using a cutoff value of 0.63. (**B**) Boxplot depicting the distribution of the original continuous Ki-67 index within each of the dichotomized groups. This plot confirms the distinct separation of the continuous Ki-67 values between the two predefined groups.

The cohort was predominantly diagnosed with esophageal squamous cell carcinoma (58/59, 98.3%), with one case of esophageal adenocarcinoma (1.7%). Ki-67 expression was evaluated by immunohistochemistry (IHC) on surgical specimens and was defined as the percentage of positively stained tumor cell nuclei in the hotspot region, reported at 5% or 10% intervals. Patients were stratified into Ki-67-high (*n* = 21) and Ki-67-low (*n* = 38) expression groups. Since the sample median was 0.6 and 11 patients had Ki-67 = 0.6 (to avoid threshold clustering), a cutoff value of 0.63 was applied. This threshold falls within the 60–70% range identified by Zhu et al. [20] as clinically relevant for esophageal cancer Ki-67 stratification. Sensitivity analyses were performed using thresholds of 0.33, 0.43, 0.53, 0.63, and 0.73 to assess robustness. The patient grouping is shown in Figure 2.

### 2.2. CT Image Acquisition and Preprocessing

All CT images were acquired using a Philips Ingenuity CT scanner. Scan parameters included: tube voltage 120 kVp; automatic tube current modulation; detector collimation 64 × 0.625 mm; pitch 1.0; field of view 350 × 350 mm; and matrix 512 × 512. Images were reconstructed at a 1 mm slice thickness. Preprocessing steps included CT value truncation: voxel values were constrained to (−200, 400) Hounsfield units (HUs) to remove extreme irrelevant signals, and voxel resampling: using linear interpolation, all images were resampled to a uniform isotropic resolution of 1 × 1 × 1 mm^3^ to minimize scanner-related spatial variations.

### 2.3. ROI Delineation and Validation

A radiation dosimetrist with >10 years of experience manually delineated three-dimensional ROIs on the non-contrast CT images using Focal software (version 4.80.00; IMPAC Medical Systems, Inc., Maryland Heights, MO, USA):

Esotarget: the solid tumor region was delineated with reference to gastroscopy-reported tumor location, and contrast-enhanced CT was used for assistance when necessary.

Eso: normal esophageal wall tissue was delineated at a site remote from the tumor.

**Figure 3:**
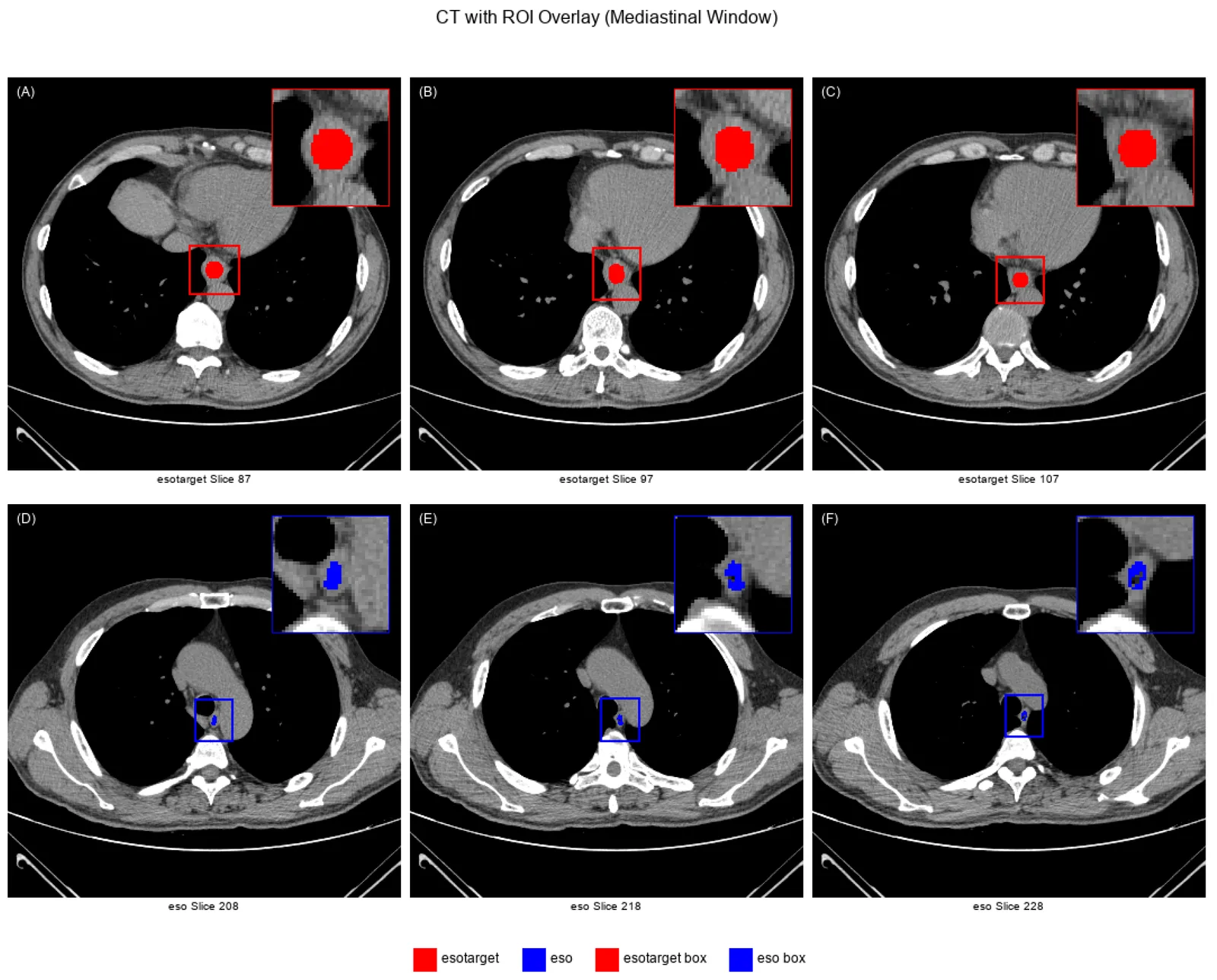
Representative examples of region of interest (ROI) delineation on non-contrast CT images. (**A**–**C**) Manual delineation of the esotarget ROI (red contour) encompassing the esophageal tumor across three different axial slices. (**D**–**F**) Manual delineation of the eso ROI (blue contour) on the normal esophageal wall, away from the tumor site, across three different axial slices. The magnified inset in the upper right corner of the composite image provides a detailed view of the delineated regions within the boxed area.

For each patient, the three-dimensional Euclidean distance between the centroids of the eso and the esotarget was computed. The mean distance was 11.3 cm (median 10.9 cm; range 5.16–24.12 cm; IQR 8.11–13.85 cm). All eso ROIs were positioned at a minimum distance of 5 cm from the tumor and were outlined on the distal normal esophageal wall, with reference to the tumor location confirmed by endoscopic reports, in order to avoid any areas of potential tumor involvement. The entire esotarget volume was not contoured; normal eso ROIs were matched to the tumor’s slice range to avoid volume disparity from differences in slice number. All ROIs were independently reviewed by a senior radiation oncologist and a thoracic surgeon to ensure anatomical accuracy. Within each ROI mask, voxels with CT values ≥ −50 HU were retained to exclude air-filled lumens. Figure 3 shows an example of a patient’s CT image overlaid with esotarget and eso ROIs.

### 2.4. Radiomics Feature Extraction and Delta-Radiomics Construction

Radiomics features were extracted from the esotarget and eso regions using pyRadiomics (v3.0.1). Extracted features mainly included three categories: shape features (14), describing the 3D morphology and size; intensity-based features (first-order statistics, 18), describing voxel value distribution; and textural features, including those from the Gray Level Co-Occurrence Matrix (GLCM, 24), Gray Level Run Length Matrix (GLRLM, 16), and Gray Level Size Zone Matrix (GLSZM, 16). Image intensities were normalized (scale = 100) with equal-width discretization (bin width = 25 HU) and B-spline interpolation. GLCM features used distance = 1 with symmetrization. To accommodate small volume structures, extraction parameters were optimized (e.g., allowing 2D ROIs, reducing minimum ROI size, and setting edge padding), which deviate from IBSI guidelines.

For each patient, the difference between esotarget and eso features was calculated to construct the delta-radiomics feature:

delta radiomics = esotarget_X − eso_X(1)

where X denotes a radiomics feature. A total of 88 delta-radiomics features were generated from the original 88 features.

### 2.5. Feature Engineering and Feature Selection

The feature preprocessing pipeline included (a) missing values: the median imputation was applied, and no missing values were found in this dataset, and (b) outliers: the interquartile range (IQR) method was used for outlier detection, and outliers were defined as values less than Q1 − 1.5IQR or greater than Q3 + 1.5IQR. Due to the high proportion of samples containing outliers (55.93%), a Winsorizing approach was used to clamp outliers to the normal range boundaries.

Feature selection was performed using minimum Redundancy Maximum Relevance (mRMR) with the MID (Mutual Information Difference) criterion, selecting k = 3 features. To prevent data leakage, feature selection was nested within each LOOCV iteration: in each of the 59 iterations, mRMR was applied only to the 58 training samples, and the selected features were then used to train the model and predict the held-out test sample. The choice of k = 3 was based on events-per-variable (EPV) considerations, with 21 positive events: EPV = 21/3 = 7.0. While an EPV ≥ 10 is a commonly cited rule of thumb for sample size, evidence suggests this criterion may be overly conservative [21]. To address the potential for model overfitting associated with a lower EPV, bootstrap internal validation was performed. Feature stability was assessed by tracking the selection frequency of each feature across all 59 LOOCV iterations.

### 2.6. Model Construction and Evaluation

Models were built using scikit-learn (v1.3.0). Three classifiers were compared: Random Forest (RF), Logistic Regression (LR), and Support Vector Machine (SVM). The Random Forest model was configured with *n*_estimators = 200, max_depth = 2, min_samples_leaf = 15, and class_weight = ‘balanced’ to account for class imbalance and prevent overfitting in the small sample. Considering the limited sample size, nested leave-one-out-cross-validation (LOOCV) [22,23] was used for model evaluation, with feature selection performed independently inside each iteration. The optimal model was selected based on the AUC and Brier score on the delta-radiomics features at the pre-specified Ki-67 threshold of 0.63.

A systematic one-factor-at-a-time (OFAT) analysis was conducted as follows: (1) model comparison (RF vs. LR vs. SVM) with fixed delta-radiomics features and a threshold of 0.63; (2) threshold sensitivity analysis (0.33, 0.43, 0.53, 0.63, 0.73) with optimal model and delta-radiomics features; (3) feature type comparison (eso vs. esotarget vs. delta radiomics) with the optimal model and a threshold of 0.63; (4) clinical variable model combined with model evaluation; (5) feature selection frequency analysis; and (6) SHAP interpretability analysis.

Performance metrics included the mean area under the ROC curve (AUC) with 95% confidence interval, accuracy, sensitivity, specificity, precision, F1-score, and Brier score. Bootstrap internal validation (2000 iterations) was performed to calculate the optimism-corrected AUC and 95% CI. Calibration curves and decision curve analysis (DCA) were generated. DeLong’s test was used for pairwise AUC comparison. Model interpretability was assessed using SHAP (SHapley Additive exPlanations; SHAP v0.45.1). For the Random Forest model, SHAP TreeExplainer was used. Global feature importance was visualized with summary plots, and individual prediction reasoning was illustrated with waterfall plots.

### 2.7. Statistical Analysis

Categorical variables were reported as numbers (percentages) and compared using the Chi-square test or Fisher’s exact test. Normally distributed continuous variables were expressed as mean ± standard deviation and compared with the independent samples *t*-test. Non-normally distributed variables were presented as the median (interquartile range) and compared with the Mann–Whitney U test. All analyses were performed using Scipy (v1.11.4). A two-sided *p*-value < 0.05 was considered statistically significant.

## 3. Results

### 3.1. Patient Baseline Characteristics

As shown in Table 1, no statistically significant differences (*p* > 0.05) were observed between the Ki-67-high and Ki-67-low expression groups in terms of age, gender, smoking history, drinking history, T stage, N stage, tumor location, or various comorbidities such as hypertension, diabetes, and cardiovascular disease. This indicates that the baseline clinical characteristics were comparable between the two groups, and subsequent differences in model performance are more likely to be attributable to radiomic features than clinical confounding factors.

### 3.2. Model Comparison

**Figure 4:**
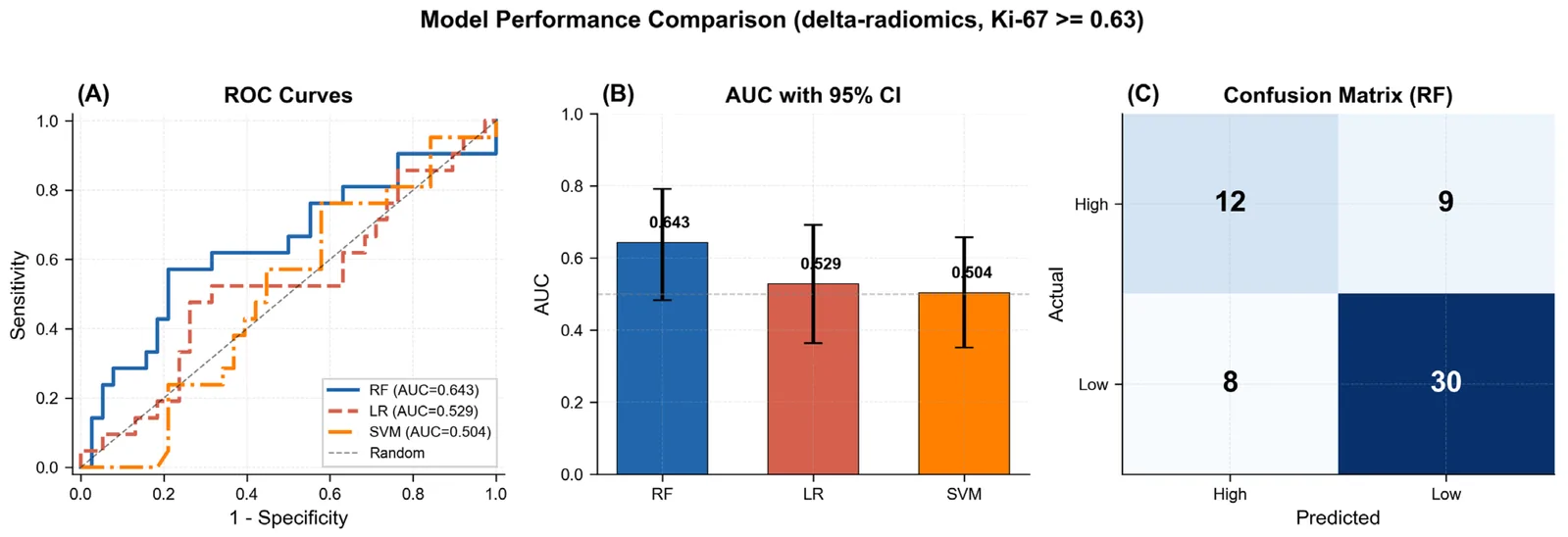
Model performance with delta-radiomics features at threshold 0.63. (**A**) ROC curves for RF, LR, and SVM. (**B**) AUC with 95% CI; RF achieves the highest AUC (0.643). (**C**) Confusion matrix of the RF model using the optimal decision threshold.

With delta-radiomics features and the pre-specified Ki-67 threshold of 0.63, three classifiers were compared using nested LOOCV (Table 2 and Figure 4). Random Forest achieved the highest AUC (0.643, 95% CI: 0.483–0.792) and the lowest Brier score (0.233) and was selected as the optimal model for all subsequent analyses. The feature selection stability was 47.5% (with 28/59 folds selecting the same feature set). Pairwise DeLong tests showed no significant differences between RF and LR (*p* = 0.544), RF and SVM (*p* = 0.462), or LR and SVM (*p* = 0.899), consistent with the overlapping confidence intervals and reflecting the limited statistical power of the small cohort.

### 3.3. Threshold Sensitivity Analysis

**Figure 5:**
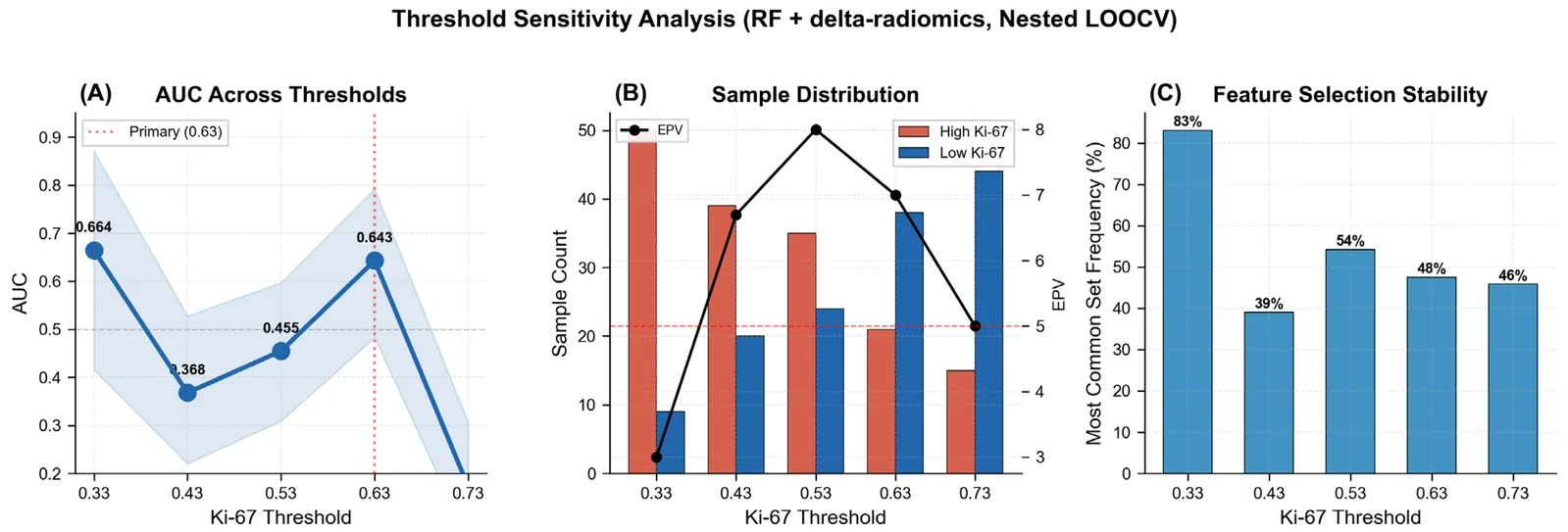
Threshold sensitivity analysis (RF, delta-radiomics features). (**A**) AUC across thresholds 0.33–0.73. (**B**) Sample distribution and EPV. (**C**) Feature-selection stability.

With the optimal RF model and delta-radiomics features, five Ki-67 thresholds were compared (Figure 5). The threshold of 0.63 achieved the best performance (AUC = 0.643) among thresholds with an adequate EPV (>=5). The threshold of 0.33 showed a higher AUC (0.664) but had severely inadequate EPV (3.0), rendering the result unreliable. The threshold of 0.73 resulted in near-random performance (AUC = 0.173), likely due to extreme class imbalance. Feature selection stability varied across thresholds, peaking at 0.33 (83.1%) and reaching its lowest value at 0.43 (39.0%).

### 3.4. Feature Type Comparison

**Figure 6:**
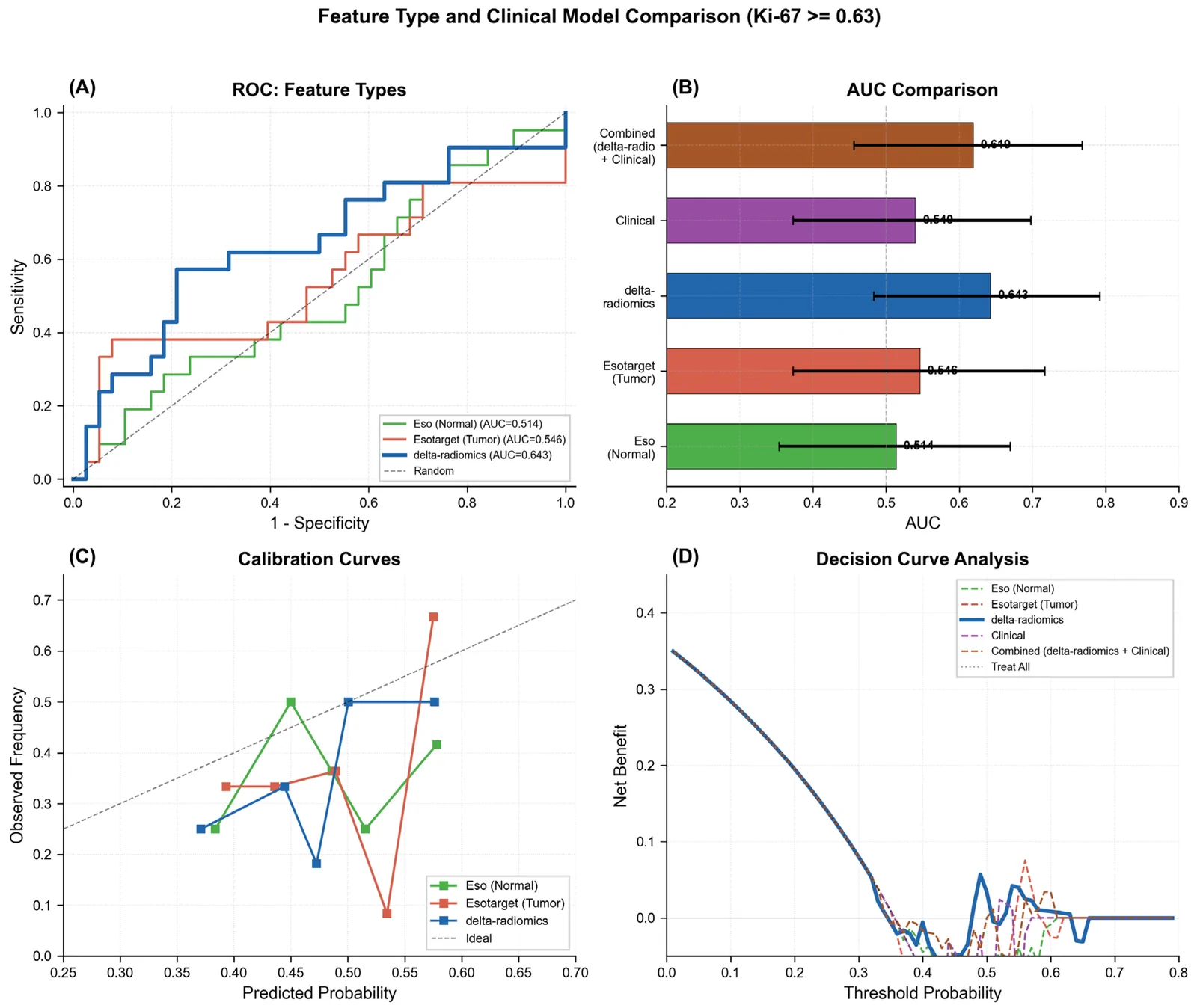
Feature type and clinical model comparison (RF, threshold 0.63). (**A**) ROC curves for delta radiomics, esotarget, and eso feature types. (**B**) AUC comparison including clinical and combined models. (**C**) Calibration curves for the three ROI-derived feature types. (**D**) Decision curves for clinical and three ROI-derived feature types.

With the optimal RF model and threshold 0.63, three feature types were compared (Figure 6). Delta radiomics (AUC = 0.643) outperformed both esotarget (AUC = 0.546) and eso (AUC = 0.514), demonstrating the incremental value of the delta-radiomics approach. Pairwise DeLong tests indicated no significant differences (delta radiomics vs. esotarget *p* = 0.607; delta radiomics vs. eso *p* = 0.493; esotarget vs. eso *p* = 0.865) attributable to the small sample size. The superior performance of delta radiomics over esotarget also provides indirect evidence for ROI delineation consistency: if the ROI for normal tissue is inconsistent (random noise), the subtraction operation amplified the noise rather than the extracted signal. Bootstrap internal validation (2000 iterations) confirmed model stability: the optimism-corrected AUC was 0.6427 (optimism = 0.0001, 95% CI: 0.483–0.792), indicating negligible overfitting.

Clinical variables alone (AUC = 0.540, 95% CI: 0.373–0.698) showed near-random performance. The combined model (delta radiomics + clinical variables, AUC = 0.619, 95% CI: 0.456–0.768) did not outperform delta radiomics alone (AUC = 0.643), suggesting that delta-radiomics features already capture the main predictive signal and clinical variables do not provide additional discriminative value in this cohort. DeLong tests confirmed that there were no significant differences (delta radiomics vs. clinical model, *p* = 0.585; delta radiomics vs. combined model, *p* = 0.898).

Calibration curves (Figure 6C) show reasonable agreement between predicted probabilities and observed outcomes for all three ROI-derived feature types, with delta radiomics exhibiting the closest alignment with the ideal calibration line. Critically, the decision curve analysis (Figure 6D) demonstrates that delta radiomics provides the highest net benefit across multiple threshold probabilities, surpassing all alternative feature types including the combined model.

### 3.5. Feature Selection Frequency

**Figure 7:**
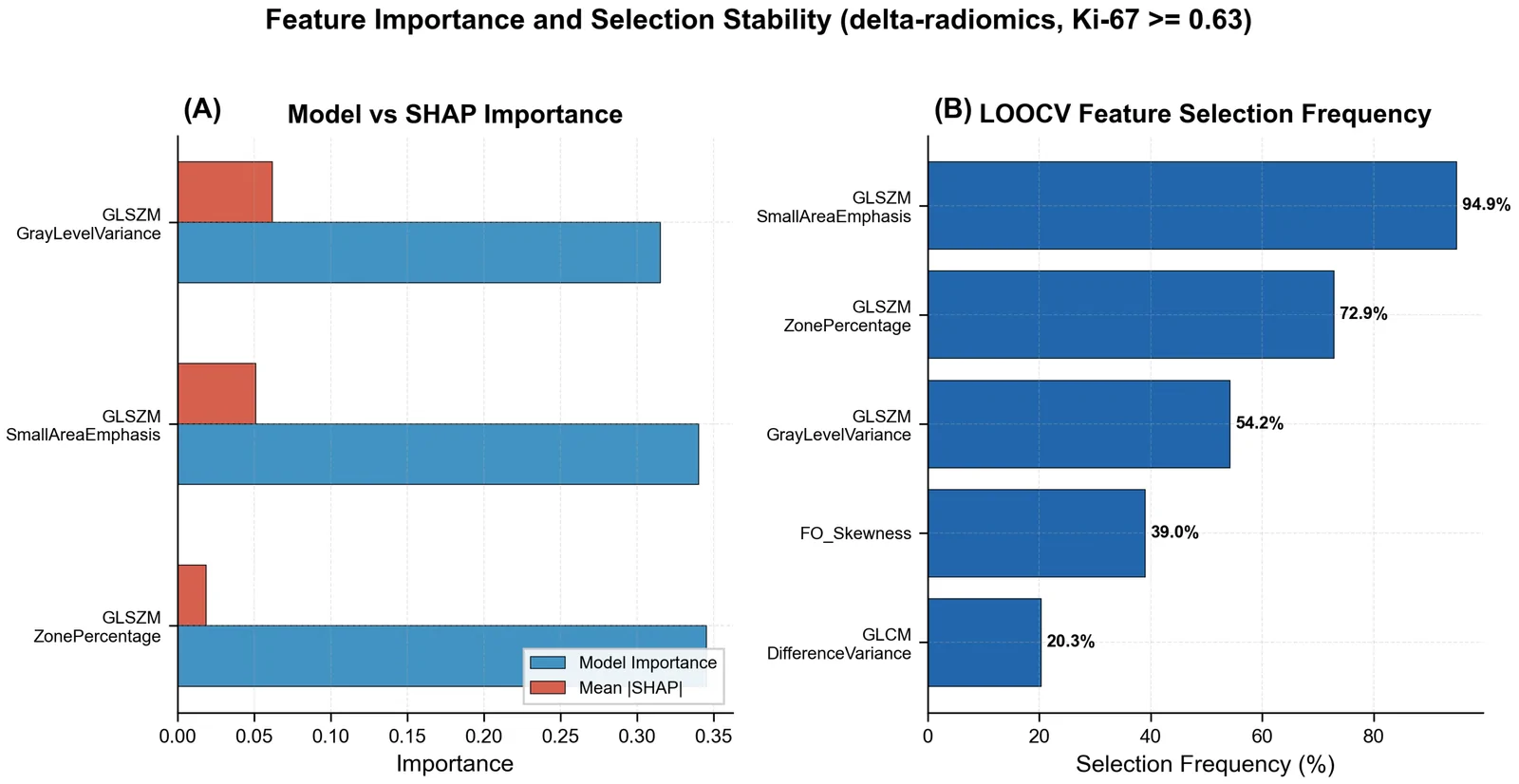
Feature importance and selection stability. (**A**) RF Gini importance versus mean |SHAP| for the top-3 features, showing concordance between the two metrics. (**B**) LOOCV selection frequency of the top features; Small Area Emphasis was selected in 94.9% of folds.

As seen in Figure 7, the mean absolute SHAP values ranked as follows: delta_glszm_GrayLevelVariance (0.062) > delta_glszm_SmallAreaEmphasis (0.051) > delta_glszm_ZonePercentage (0.019). GrayLevelVariance, reflecting gray-level heterogeneity in the size-zone matrix, was the most influential feature driving the model’s predictions. SmallAreaEmphasis, characterizing the distribution of small-volume regions, was consistently identified as important by both SHAP and LOOCV selection frequency, validating its biological relevance to tumor proliferative activity.

Across 59 LOOCV iterations, the most frequently selected features were: delta_glszm_SmallAreaEmphasis (56/59, 94.9%), delta_glszm_ZonePercentage (43/59, 72.9%), and delta_glszm_GrayLevelVariance (32/59, 54.2%). Notably, SmallAreaEmphasis was identified as a key feature across multiple analytical approaches, demonstrating cross-model feature stability. The overall feature set stability was 47.5% (where 28/59 folds selected the same combination of three features: GrayLevelVariance, SmallAreaEmphasis, and ZonePercentage), which was the single most frequent feature triple.

### 3.6. SHAP Interpretability Analysis

**Figure 8:**
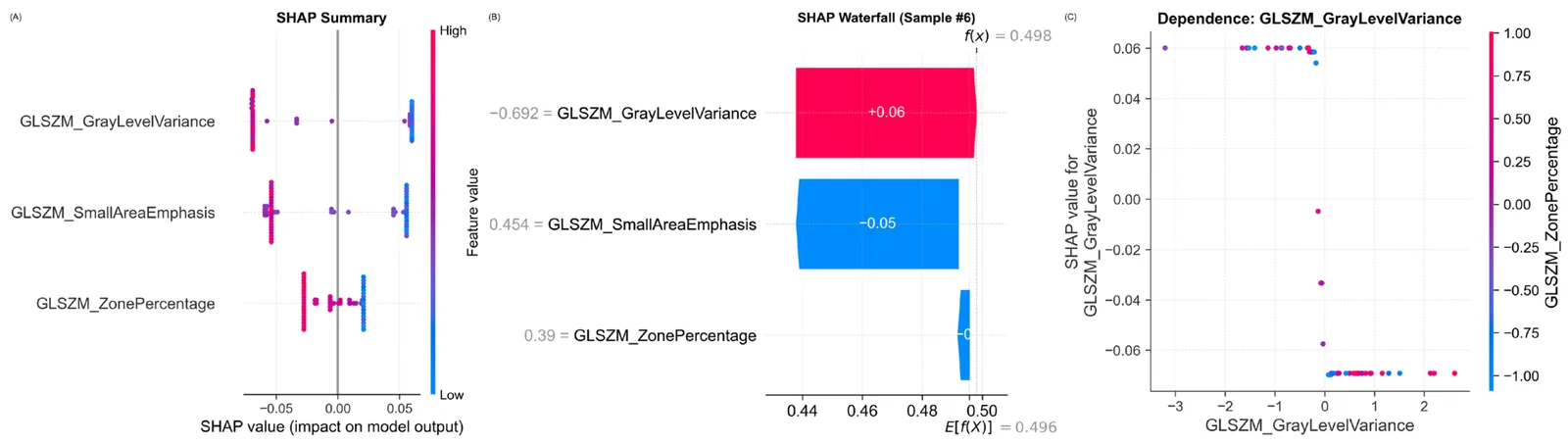
SHAP interpretability of the RF model. (**A**) SHAP summary beeswarm for the top-3 features, showing direction and magnitude of impact. (**B**) Waterfall plot for a representative sample, decomposing the prediction into additive feature contributions. (**C**) Dependence plot for GrayLevelVariance, showing its interaction pattern with the predicted outcome.

Figure 8 provides a SHAP-based interpretability analysis of the optimal delta-radiomics + Random Forest model. The SHAP summary plot (Figure 8A) reveals the global importance and impact direction of the three most influential features. GrayLevelVariance exhibits the highest mean absolute SHAP value, indicating that gray-level intensity heterogeneity within the GLSZM is the strongest driver of the model’s predictions. High GrayLevelVariance values (shown in red) push predictions toward the Ki-67-high class, which is biologically consistent with the notion that rapidly proliferating tumors exhibit greater disorganization in tissue density. SmallAreaEmphasis, the second most influential feature, characterizes the prevalence of small homogeneous zones within the tumor; elevated values reflect fine-scale textural heterogeneity associated with aggressive tumor biology. ZonePercentage shows a comparatively lower SHAP impact, suggesting it serves as a stable but secondary contributor. The SHAP waterfall plot (Figure 8B) deconstructs an individual prediction for a representative patient classified as Ki-67-low, illustrating how each feature incrementally adjusts the prediction from the base value. The dependence plot (Figure 8C) visualizes the relationship between GrayLevelVariance and its SHAP value, confirming a monotonic trend: higher GrayLevelVariance consistently increases the predicted probability of Ki-67-high status. Together, these analyses demonstrate that the model’s predictions are driven by texture features reflecting intratumoral heterogeneity—a hallmark of proliferative activity—providing biologically plausible evidence that the delta-radiomics signature captures meaningful information related to Ki-67 expression rather than spurious statistical associations.

## 4. Discussion

This exploratory study developed and validated a Random Forest model based on preoperative CT-derived delta-radiomics features for the non-invasive prediction of Ki-67 expression in esophageal cancer. Using nested LOOCV to eliminate data leakage, the model achieved an AUC of 0.643 (95% CI: 0.483–0.792), supporting the feasibility of using delta-radiomics features to assess tumor proliferative activity, while acknowledging the moderate performance.

A key finding of this study is the effectiveness of the delta-radiomics strategy. By computing feature differences between the tumor and paired normal tissue, this method effectively reduced noise from inter-individual anatomical and physiological variations. The delta-radiomics model (AUC = 0.643) outperformed both the esotarget model (AUC = 0.546) and the eso model (AUC = 0.514), demonstrating that the subtraction approach extracts a predictive signal beyond what is available from tumor features alone. The superior performance of delta radiomics over esotarget also provides indirect evidence for ROI delineation consistency: if the normal tissue ROI introduced random noise, the subtraction would degrade rather than improve performance. Furthermore, the high feature selection frequency (SmallAreaEmphasis: 94.9%) across LOOCV iterations indicates that the selected features are robust to sample variation, further supporting delineation consistency. This three-layer model comparison (eso → esotarget → delta radiomics) thus provides indirect validation of ROI delineation consistency: the monotonic improvement from eso (0.514) and esotarget (0.546) to delta radiomics (0.643) would not be observed if the ROI for normal tissue introduced unstructured noise.

The three features selected most frequently spanned the texture domain, all originating from the Gray Level Size Zone Matrix (GLSZM). SmallAreaEmphasis, selected in 94.9% of LOOCV iterations, characterizes the distribution of small-volume zones within the tumor and reflects fine-scale textural heterogeneity. This feature has been identified as an important predictor of molecular marker expression in other gastrointestinal malignancies; Wei et al. identified SmallAreaEmphasis as a key predictor of HER2 status in bladder cancer [24]. In esophageal cancer, GLSZM-derived texture features have been shown to capture intratumoral heterogeneity associated with staging and treatment response [25,26]. The predominance of GLSZM features in our model is biologically plausible: these features capture the spatial distribution of homogeneous regions at multiple scales, reflecting the disorganized tissue architecture that accompanies rapid cellular proliferation. The structural complexity captured by these textural features aligns with the cellular proliferation and tumor heterogeneity represented by Ki-67 [27]. Notably, SmallAreaEmphasis was consistently identified as a key feature across multiple analytical approaches, demonstrating cross-method feature stability.

From a translational perspective, this study provides proof-of-concept for utilizing imaging to non-invasively assess Ki-67 expression. Future potential applications may include imaging-based monitoring of tumor proliferative activity, pending validation in prospective multi-center studies. Conventional efficacy assessments such as RECIST criteria are often limited by delayed timelines and imprecision [28,29]. If future prospective studies confirm that Ki-67-associated delta-radiomics features change systematically after treatment, routine CT could potentially serve as an “imaging biopsy.”

We acknowledge several limitations. First, the single-center retrospective design and small sample size (*n* = 59, 21 positive events) limit generalizability. Second, no external validation was performed; the Bootstrap optimism of 0.0001 supports internal consistency but cannot replace external validation. Third, the model performance (AUC = 0.643) is moderate and leaves substantial room for improvement. Finally, the Ki-67 threshold of 0.63, while supported by the 60–70% range identified in the esophageal cancer literature [20], was based on the sample median and lacks established clinical significance.

## 5. Conclusions

This exploratory study developed a Random Forest model utilizing delta-radiomics features extracted from preoperative non-contrast CT images to predict Ki-67 expression status in esophageal cancer. The model achieved moderate discriminatory performance (AUC = 0.643), with delta radiomics outperforming conventional tumor-only radiomics (AUC = 0.546) and clinical variables (AUC = 0.540). Key texture features, particularly SmallAreaEmphasis and GrayLevelVariance, were consistently identified as important predictors. This study is hypothesis-generating; external validation in independent multi-center cohorts with larger sample sizes is required before clinical application.

## Figures and Tables

**Table 1:** Comparison of baseline characteristics between patients with high and low Ki-67 expression.

Characteristic	Ki-67-High	Ki-67-Low	*p*-Value	Test
Age (years)	64.3 ± 9.0	65.8 ± 8.2	0.530	Student *t*-test
Gender			1.000	Fisher exact
Male	18 (85.7%)	33 (86.8%)		
Female	3 (14.3%)	5 (13.2%)		
Smoking History			0.707	Chi-square
Yes	12 (57.1%)	25 (65.8%)		
No	9 (42.9%)	13 (34.2%)		
Alcohol History			0.302	Chi-square
Yes	9 (42.9%)	23 (60.5%)		
No	12 (57.1%)	15 (39.5%)		
T Stage			0.788	Mann–Whitney U
1	2 (9.5%)	4 (10.5%)		
2	8 (38.1%)	15 (39.5%)		
3	11 (52.4%)	18 (47.4%)		
N Stage			0.678	Mann–Whitney U
0	8 (38.1%)	17 (44.7%)		
1	8 (38.1%)	12 (31.6%)		
2	4 (19.0%)	9 (23.7%)		
3	1 (4.8%)	0 (0.0%)		
Location			0.569	Fisher exact (simulated)
Upper	1 (4.8%)	3 (7.9%)		
Middle	9 (42.9%)	17 (44.7%)		
Lower	10 (47.6%)	18 (47.4%)		
Hypertension	6 (28.6%)	16 (42.1%)	0.454	Chi-square
Diabetes	0 (0.0%)	6 (15.8%)	0.080	Fisher exact
Cardiovascular Disease	0 (0.0%)	2 (5.3%)	0.534	Fisher exact
Cancer History	1 (4.8%)	2 (5.3%)	1.000	Fisher exact
Any Comorbidity	7 (33.3%)	22 (57.9%)	0.125	Chi-square
Neoadjuvant Therapy	2 (9.5%)	7 (18.4%)	0.469	Fisher exact

**Table 2:** Model comparison using delta-radiomics features at a Ki-67 threshold of 0.63.

Model	AUC	95% CI	Accuracy	Sensitivity	Specificity	F1	Brier
RF (optimal)	0.643	0.483–0.792	0.712	0.571	0.789	0.585	0.233
LR	0.529	0.363–0.692	0.644	0.476	0.737	0.488	0.266
SVM	0.504	0.351–0.657	0.542	0.762	0.421	0.542	0.252

## Data Availability

The datasets generated and/or analyzed during the current study are available from the corresponding authors on reasonable request.

## Abbreviations

The following abbreviations are used in this manuscript:

ROC	Receiver operating characteristic
AUC	Area under the curve
CI	Confidence interval
CT	Computed tomography
DECT	Dual-energy computed tomography
GLCM	Gray level co-occurrence matrix
GLRLM	Gray level run length matrix
GLSZM	Gray level size zone matrix
HUs	Hounsfield units
IQR	Interquartile range
LOOCV	Leave-one-out cross-validation
MRI	Magnetic resonance imaging
ROI	Region of interest
SHAPs	SHapley Additive exPlanations
